# Advanced and Rationalized Atomic Force Microscopy Analysis Unveils Specific Properties of Controlled Cell Mechanics

**DOI:** 10.3389/fphys.2018.01121

**Published:** 2018-08-17

**Authors:** Guido Caluori, Jan Pribyl, Martin Pesl, Jorge Oliver-De La Cruz, Giorgia Nardone, Petr Skladal, Giancarlo Forte

**Affiliations:** ^1^International Clinical Research Center of the St. Anne's University Hospital Brno (FNUSA-ICRC), Interventional Cardiac Electrophysiology, Brno, Czechia; ^2^Central European Institute of Technology of Masaryk University, Nanobiotechnology, Brno, Czechia; ^3^Department of Biology, Faculty of Medicine, Masaryk University, Brno, Czechia; ^4^First Department of Internal Medicine/Cardioangiology, St. Anne's Hospital, Masaryk University, Brno, Czechia; ^5^International Clinical Research Center of the St. Anne's University Hospital Brno (FNUSA-ICRC), Center for Translational Medicine, Brno, Czechia; ^6^Department of Biomaterials Science, Institute of Dentistry, University of Turku, Turku, Finland

**Keywords:** atomic force microscopy, cell biomechanics, BEEC, force mapping, mechanical modeling, stiffness tomography, Hippo pathway, mechanotransduction

## Abstract

The cell biomechanical properties play a key role in the determination of the changes during the essential cellular functions, such as contraction, growth, and migration. Recent advances in nano-technologies have enabled the development of new experimental and modeling approaches to study cell biomechanics, with a level of insights and reliability that were not possible in the past. The use of atomic force microscopy (AFM) for force spectroscopy allows nanoscale mapping of the cell topography and mechanical properties under, nearly physiological conditions. A proper evaluation process of such data is an essential factor to obtain accurate values of the cell elastic properties (primarily Young's modulus). Several numerical models were published in the literature, describing the depth sensing indentation as interaction process between the elastic surface and indenting probe. However, many studies are still relying on the nowadays outdated Hertzian model from the nineteenth century, or its modification by Sneddon. The lack of comparison between the Hertz/Sneddon model with their modern modifications blocks the development of advanced analysis software and further progress of AFM promising technology into biological sciences. In this work, we applied a rationalized use of mechanical models for advanced postprocessing and interpretation of AFM data. We investigated the effect of the mechanical model choice on the final evaluation of cellular elasticity. We then selected samples subjected to different physicochemical modulators, to show how a critical use of AFM data handling can provide more information than simple elastic modulus estimation. Our contribution is intended as a methodological discussion of the limitations and benefits of AFM-based advanced mechanical analysis, to refine the quantification of cellular elastic properties and its correlation to undergoing cellular processes *in vitro*.

## Introduction

The determination of the biomechanical properties of cells and their surrounding extracellular matrix (ECM) unveils fundamental insights to understand the development and the features of healthy and pathological conditions (Yim and Sheetz, 2012). Cellular mechanics is a dynamic process originating from the disposition and interaction of the cytoskeletal proteins (mainly actin microtubules and intermediate filaments) (Ahmed et al., 2015; Huber et al., 2015), and by its coupling to the cell nucleus (Li et al., 2014). The appropriate organization and composition of the cytoskeleton allows cells to proceed in their cell cycle (Nakaseko and Yanagida, 2001; Heng and Koh, 2010) and to adapt to environmental changes [e.g., drugs (Li et al., 2015), ECM composition (Klaas et al., 2016), applied forces and substrate stiffness Discher et al., 2005] via mechano-transduction pathways (Huang et al., 2004). A common regulation mechanism resides in the formation of focal adhesions between the actin-integrin transmembrane complex and target ECM binding sites (Huang et al., 2004). Focal adhesion formation induces the upregulation of different biomolecular cascades, including the Hippo pathway through YAP and TAZ transcription factors (Benham-Pyle et al., 2015). The alteration of cytoskeletal homeostasis is common in many pathological processes. For instance, in cancer metastasizing, actin fibers are remodeled so that stiffness can drop sensibly, thus providing sufficient motility to invade neighboring tissues (Cross et al., 2007). Skeletal and cardiac muscle myotubules alteration can lead to impaired excitation-contraction coupling in dystrophinopathies (Kerr et al., 2015). Endothelial cells can respond to altered fluid dynamics and increased shear-stress with hypertrophy and tissue stiffening (Lu and Kassab, 2011).

**Figure 1:**
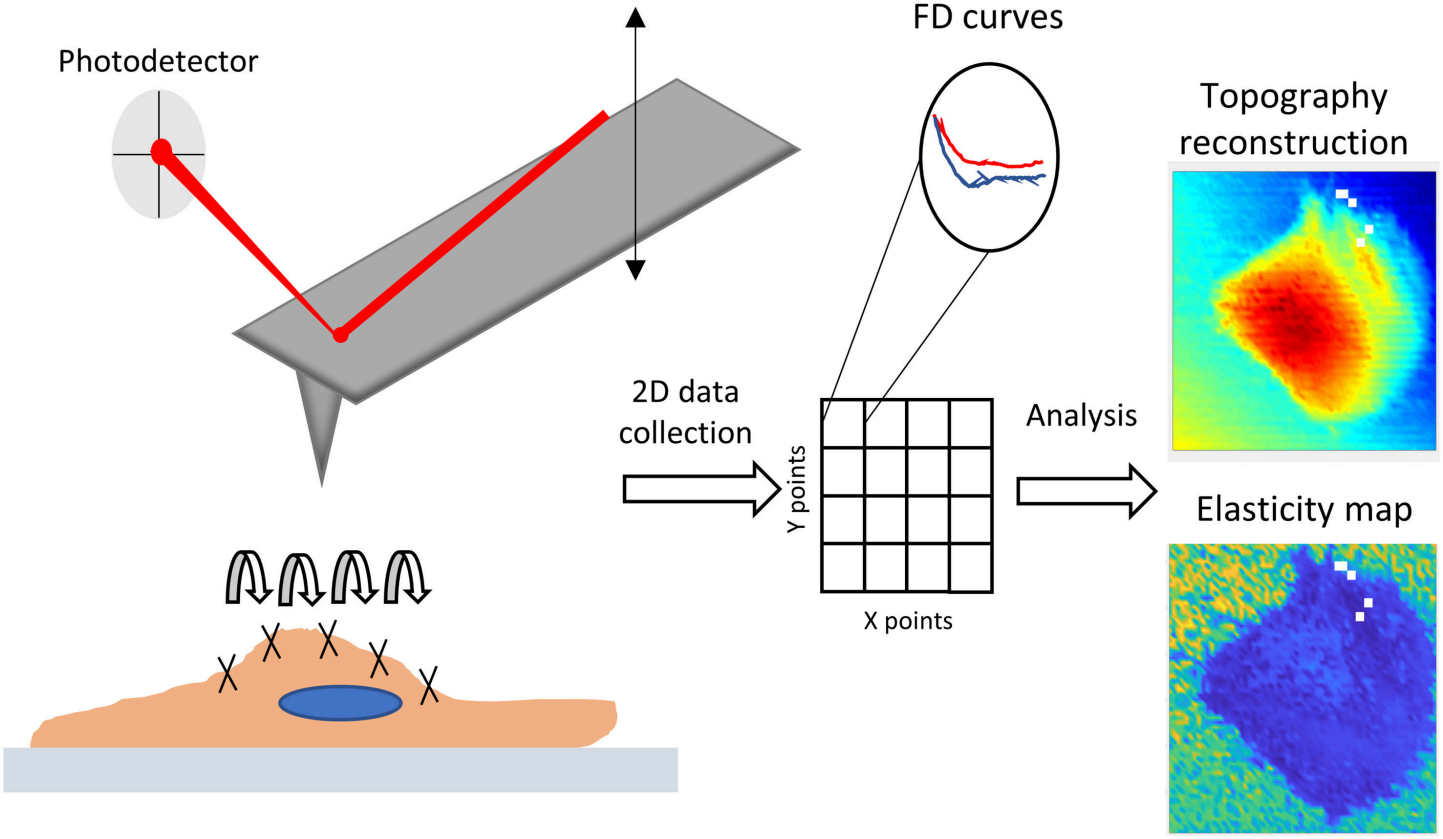
Graphical depiction of AFM force probing, mapping and typical results used in this work.

It has become clear that, to fully understand biomechanical features, a multiscale approach is indispensable (Bausch and Kroy, 2006). A characterization of the biomechanical molecules is a paramount step, for which immunocytochemistry and live staining microscopy are powerful approaches to visualize cytoskeletal spatiotemporal organization. Nevertheless, techniques based on molecular approaches still struggle to establish methods for quantitative evaluation (Alhussein et al., 2016). At the cellular level, most of the molecular complexity can be ignored to rely on established continuum-based mechanical models (Huang et al., 2004). In this way, it is possible to describe biomechanical features in terms of stresses (forces) and strains (displacement) (Moeendarbary and Harris, 2014), linking them through concentrated parameters such as elastic (or Young's) modulus, and loss modulus (Guz et al., 2014). Force-displacement relationships can be measured through passive methods (e.g., particle tracking techniques) (Wirtz, 2009), or active ones, such as optical tweezers (Ayala et al., 2016), magnetic beads (Marjoram et al., 2016), polymeric micropillars (Hanson et al., 2015), or depth sensing instrumentation (Oyen, 2015). Among the latter typology, atomic force microscopy (AFM) applies precisely controlled forces through a nanosized indenter placed on a cantilever, whose position is defined by a piezoelectric actuator. The sweeping vertical motion of the AFM tip allows the recording of force-distance curves (FDC) (Butt et al., 2005), which contain information on cellular stiffness, and, through model-based interpretation, on the related viscoelastic parameters. FDCs can be performed pointwise on a 2D grid, creating a so-called force-volume map. The typical process and results of probing mechanical properties with AFM force mapping are shown in Figure 1. The attractiveness of AFM for biological samples is well-known, and it resides in the non-destructive nature of the technique, the minimal preparation necessary to analyze biological samples in liquids, and the nanometric resolution of both applied force and measured indentations (Müller and Dufrêne, 2008). The technique is easily integrated with optical microscopy (Geisse, 2009; Cascione et al., 2017), and scalable from cell population to subcellular domains. So far, AFM has been applied extensively in the field of biomechanical studies (Engler et al., 2007; Pesl et al., 2016; Li et al., 2017; Alcaraz et al., 2018; Borin et al., 2018; Zemła et al., 2018). Despite its potential, the heterogeneous methodology and simplistic models applied to data interpretation can lead to biased results. These facts call for a better understanding and a rationalized use of the methods, ideally aiming to a procedural standardization among AFM users (Schillers et al., 2017). For these reasons, in this work we show how the rationalized use of different AFM methodologies alone, with standardized data acquisition, can increment the information content for the understanding of environmental changes or gene-editing effects on cellular biomechanics.

## Materials and methods

### Cell culture

All the cells analyzed in this work were prepared as previously described by Nardone et al. (2017). Briefly, ASC52telo, hTERT immortalized adipose-derived mesenchymal stem cells (AD-MSC, ATCC SCRC-4000) were purchased from American Type Culture Collection (ATCC, Manassas, USA). CAL51 cancer cell lines were a gift of Dr L. Krejčí (Department of Biology, Masaryk University, Brno, Czech Republic). The cells were cultured in high glucose DMEM medium (DMEM high Glucose 4.5 gl^−1^, Lonza, Basel, Switzerland) supplemented with 10% fetal bovine serum, 2 mM L-glutamine and 100 U ml^−1^ penicillin/streptomycin. For the mechanical trials following down-regulation of the Hippo pathway, responsible for promoting proliferation and survival of breast cancer cells (Shi et al., 2015), YAP-deficient CAL51 lines (CAL51-C3) were produced by CRISPR/Cas9 technology. Guiding RNA was designed to hit exon 1 of *YAP1* gene, which is common to all nine *YAP1* splicing variants.

Cells for AFM experiments were seeded on fibronectin (FN)-coated 34 mm polystyrene (PS) Petri dishes (TPP, Trasadingen, Switzerland), FN-coated glass coverslips, and poly-L-lysine-coated (PLL) PS dishes. All the samples were let to attach for 2 days before medium change and force-mapping. The cells were kept in a 5% CO_2_, 37°C incubator prior to experiments. Single 2 ml vials filled with medium were kept open in incubator to provide physiological pH at the start of every mapping.

### Atomic force microscopy

Force -volume maps were recorded using a JPK NanoWizard 3 (JPK, Berlin, Germany) AFM system, embedded in an inverted light microscope Olympus IX-81. Scanning range of the AFM head was 100–100–15 μm, in X-Y-Z axis, respectively. Non-coated silicon nitride AFM cantilevers Hydra 2R-100N (AppNano, California, USA) were used for all the experiments. This cantilever model presents a pyramidal silicon tip (half angle to edge 18°) and has nominal spring constant 0.01 N m^**−1**^. The system was calibrated before each experiment with the following procedure. The cantilever chip was mounted on the scanner head and exposed to sterilizing UVC light for 30 min. Then, the scanner head was placed on top of a clean Petri dish filled with 2 ml double-distilled water. The dish was encased in a Petri dish heater (JPK, temperature set at 37°C), and equipped with a fluid exchange needle system. The cantilever was immersed in liquid, the laser spot was aligned at its free extremity, and the system was let to stabilize for 15 min. The laser photodetector was centered, and the cantilever tip was approached to the Petri dish surface. A single FDC (15 μm vertical sweep, 3 s extension time, z-closed loop enabled) was performed to estimate the system deflection sensitivity. Then, the cantilever was withdrawn at least 1 mm away from the surface, to allow the estimation of the spring constant by the thermal noise method (Butt and Jaschke, 1995). The relative error of the calibration method was estimated at 6.72%, comparable with other error sources like the point of contact detection (6.21% as measured on rigid calibrating surface).

After calibration, the water-filled Petri dish was substituted with one containing the sample. No thermal drift was observed in time, due to reduced reflective coating of the cantilever. Microscope objective with 10x magnification was used to find appropriate cell-covered regions. Force maps were performed over an area of 100x100 μm following a square 64x64 grid. For all the experiments, the maximal setpoint relative to the baseline was 1 nN, the indentation speed was 30 μm s^−1^, and the sampling frequency was 2 kHz. The average mapping time was 45 min, and after each force map, the dish medium was exchanged with fresh medium from incubator. We scanned single cells for AD-MSC or multicellular colonies for CAL51 (both C3 or wild type, WT).

### Biomechanical data interpretation

The obtained force maps were processed in a Matlab R2017b graphic user interface (Mathworks, Natick, Massachussets, USA), using state-of-the-art algorithms and mechanical models. Specifically, the approaching part of each set of FDC was detrended to set the non-contact portion of the curve to zero. Subsequently, the point of contact between the sample and the cantilever tip was assessed by trial point followed by power law fitting. The trial point was obtained by detection of a force bigger than four times the standard deviation of the first 25% of the curve. The final contact point was obtained by intersecting a linear fitting in the non-contact region with a quadratic fitting in the contact region (Cogollo et al., 2011). The quadratic fit has the form:

(1) $$P=\alpha {\left(h-h_{c}\right)}^{m}$$

Where *P* is the force, *h* is the position of the vertical piezoelectric crystal, *h*_*c*_ is the piezo position at the contact point, α is a constant containing properties of the indenter geometry and the sample elastic modulus, and *m* = 2 for conical and pyramidal indenters (Merle et al., 2014). Once the point of contact is found, load-indentation curves are calculated by subtracting the cantilever deflection from the displacement (Cogollo et al., 2011), with the formula:

(2) $$\begin{array}{l} \delta =h^{*}-d \end{array}$$

Where δ is the sample indentation, h^*^ is the position of the vertical piezoelectric actuator after contact and *d* the deflection of the cantilever. The experimental points of the calculated load-indentation curves were fitted by a smoothed spline, and values of elastic modulus were finally extracted at different indentation levels using three different mechanical models. Maximal indentation admitted was 500 nm. The evaluation of the Young's modulus at multiple indentation levels allowed us to construct stiffness tomography maps (Roduit et al., 2009). Indentation speed was kept constant; thus, we could ignore the viscous component of the cell biomechanics during comparisons. The implemented models will be briefly explained.

#### Sneddon and bilodeau model

The Sneddon's model follows the classical Hertzian theory for indentation of an infinite, purely elastic half-space with a uniformly defined conical indenter (Poon et al., 2008) or body of revolution (Sneddon, 1965). For pyramidal indenters, a more realistic model for AFM probing tips, a corrected solution was introduced by Bilodeau (1992). The elastic modulus is obtained by the following equation:

(3) $$\begin{array}{l} E=\frac{P\left(1-\nu^{2}\right)}{0.7453\text{ tan }\theta \delta^{2}} \end{array}$$

where ν is the Poisson's ratio (0.5 for incompressible materials) and θ is the half angle of the pyramidal indenter.

#### Oliver and pharr model

The method of Oliver and Pharr is a modification of the Sneddon's theory which accounts for the changing load-indentation slope, due to the indentation-dependent contact area of an axisymmetric indenter (VanLandingham et al., 2001; Poon et al., 2008). No hysteresis between non-contact region of the curves or plastic deformation was observable during multiple indentations. The modulus is calculated by:

(4) $$\begin{array}{l} E=\frac{S\;\left(1-\nu^{2}\right)\sqrt{\pi }}{2\sqrt{A}} \end{array}$$

where *A* = δ^2^π*(tan*θ*)*^2^ is the projected area of the pyramidal indenter, and *S* is the derivative of *P* over δ.

#### Bottom effect cone correction model

The bottom effect cone correction (BECC) model is a modification of Sneddon's model which amends the assumptions of infinite half-space, by considering the sample height (Gavara and Chadwick, 2012). The elastic modulus is calculated by:

(5) $$\begin{array}{l} E=\;\frac{3\pi P}{8\text{ tan }\theta }{\left\{1+1.7795\frac{2\text{ tan }\theta }{\pi }\frac{\delta }{H}+16\left(1.7795^{2}\right){\text{tan }\theta }^{2}\frac{\delta^{2}}{H^{2}}\right\}}^{-1} \end{array}$$

where the *H* is the local height of the sample. It is worth mentioning that if we exclude the term on the right side between brackets, we obtain the Sneddon's elastic modulus. In the presented experiments, *H* is extracted after calculating a flattened topographical map, in order to avoid biases due to the sample tilt.

A graphical description of the analysis algorithm is shown in Supplementary Materials.

### Statistical methods

Statistical analysis was performed in Prism 5.0 (GraphPad Software, La Jolla, CA-USA) or Matlab. All the datasets were firstly evaluated for normality distribution by Kolmogorov-Smirov test, and, once passed, were presented as mean± *SEM*. For the model comparison, repeated-measures one/two-way analysis of variance (ANOVA) with Bonferroni posttest were used. For the evaluation of the substrate effect, Welch's ANOVA test followed by Games–Howell test was performed, due to the datasets being non-homoscedastic. For the evaluation of YAP genetic knockout effect, two-way ANOVA with Bonferroni posttest and Student's *t*-test with Welch's correction were used. Statistical significance was accepted with *p*-values smaller than 0.05. Sample sizes were based on previously published experiments, in which statistical differences were identified.

## Results

### Effect of the model selection

**Figure 2:**
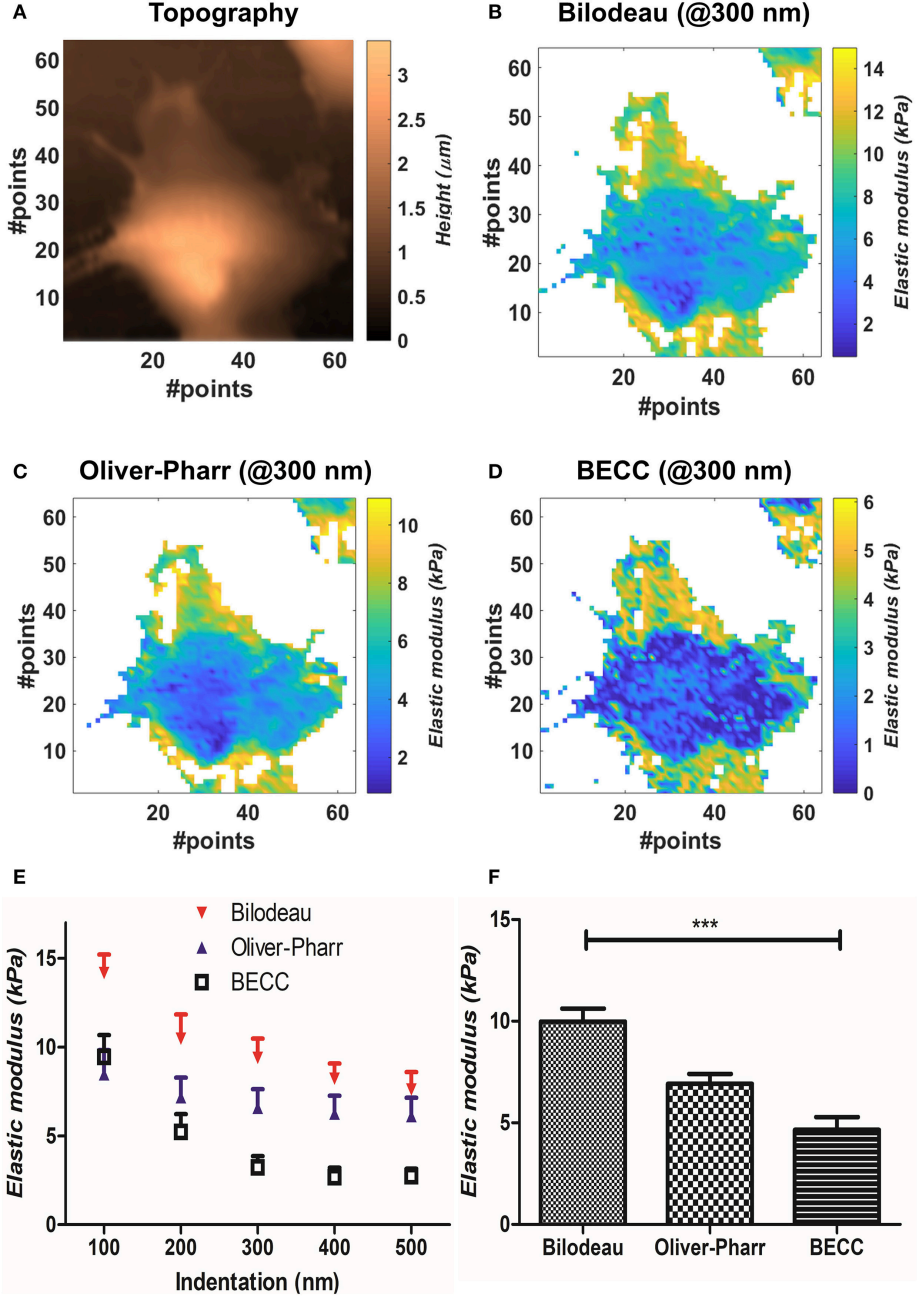
Results obtained by mechanical model comparisons. **(A)** Sample topographical map reconstructed by detection of contact points. **(B–D)** Elastic modulus distribution at 300 nm for the three selected models. It is appreciable how BEEC model shows marked differences between cellular regions. **(E)** Elastic modulus trends at different indentations, according to each selected model (mean±SEM). The values are calculated during two-way ANOVA statistical test. **(F)** Bar plot of the mean elasticity values over all the analyzed volume, according to the mechanical model used (mean± SEM). Statistical significance is given by one-way repeated measures ANOVA with Bonferroni posttest (***p < 0.0001).

We compared the selected mechanical models on well-spread cellular samples of AD-MSCs (i.e., where the substrate was clearly identifiable), cultivated on a standard fibronectin-coated Petri dish. Starting from the obtained map of contact points (Figure 2A), the appropriate model was used to estimate the Young's modulus over the whole force map, at five different indentation levels (100–200–300–400–500 nm). We performed two-way repeated measures ANOVA, with matching on the indentation levels, to assess whether using different models on the same samples will lead to statistically different results (Figure 2E). Across the measured samples (*N* = 5), the model choice accounted for largely significant difference (*p* < 0.0001). The test returned also a significant justification for matching (*p* < 0.0001), together with an accountable difference due to the different indentation levels (*p* = 0.0027). Bonferroni's posttest showed at which indentations the models start to diverge: Bilodeau's and Oliver-Pharr's models differ before 400 nm; BEEC and Oliver-Pharr's show this tendency after 300 nm; BEEC and Bilodeau's models differ significantly at each fixed indentation. For more details on the significance levels, the reader is addressed to Supplementary Materials. We then performed repeated measures one-way ANOVA using all the values calculated across the cell volumes, categorizing them only by the model used. The test returned significant difference between the datasets and significant justification for matching (*p* < 0.001, Figure 2F).

We then compared the results obtainable by the selected mechanical models. Globally, the Bilodeau's model led to the highest overestimation of the elastic modulus (9.9 ± 2.6 kPa), when compared to the values obtained by Oliver-Pharr's (6.92 ± 0.96 kPa) and BECC (4.7 ± 2.9 kPa). The BECC model showed the highest coefficient of variation across the samples and the indented volume (61.5%), followed by Bilodeau's model (26.0%) and Oliver-Pharr's (13.9%). When considered at the same indentation level, Bilodeau's and Oliver-Pharr's showed smooth distribution of the elastic modulus over the cell body, whereas BECC showed marked and detailed differences between softer and harder regions (Figures 2B–D). We observed how all the BEEC and Oliver-Pharr's models stabilized at 300 nm of indentation (slope of the mean elastic modulus 0.34 and 0.54 Panm^−1^, respectively), whilst the Bilodeau's model is still affected by depth-dependent trend (1.17 Panm^−1^). The comparative model trends are shown in Figure 2E.

### Effect of the substrate

**Figure 3:**
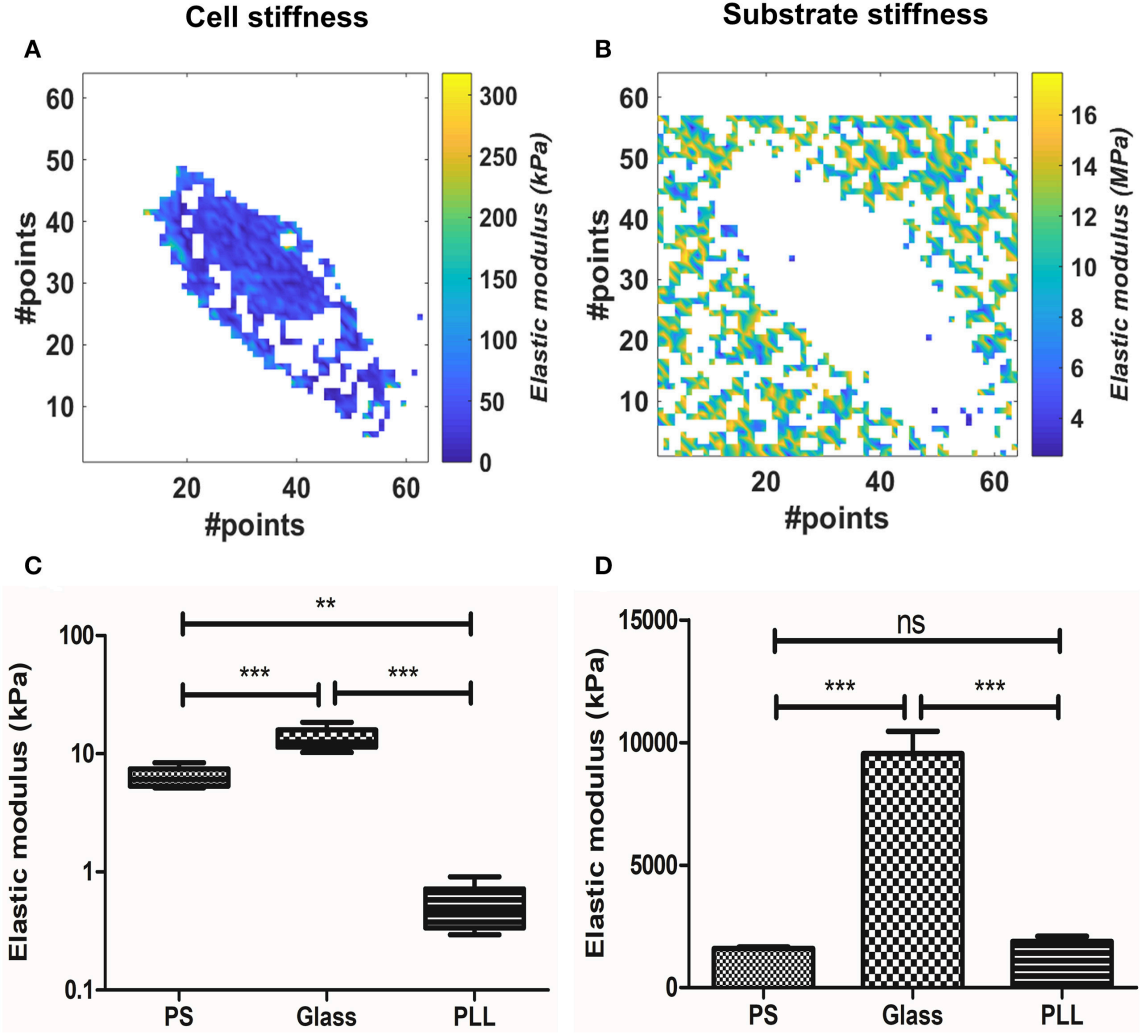
Analysis of the substrate effect on cellular stiffness, using BEEC model. The pixels in white represent data out of range (mostly due to faulty curve to be evaluated). **(A)** Isolated elasticity values corresponding to the area of a cell adhered on fibronectin-coated glass. **(B)** Relative isolated elasticity values corresponding to the glass culture substrate. **(C)** Boxplot of the cellular stiffness (line represents the median and whiskers indicate data range). **(D)** Bar plot of the correlated substrate stiffness (mean± SEM). Different levels of statistical difference were obtained comparing 5 independent samples per group, with Welch's ANOVA and Games-Howell posttest. (ns, non-significant; **p < 0.01; ***p < 0.0001).

We evaluated the effect of substrate stiffness on the elastic modulus at 300 nm for AD-MSC single cells force maps. We used the BECC model to exploit its higher discrimination capabilities and stability, given the fact that the sample substrate was clearly identifiable across the force map. Figures 3A,B show representative results of selected points belonging either to the probed cell or to the culture substrate. Cells plated on FN-coated Petri dish (polystyrene, PS, according to Figures 3C,D), FN-coated glass (Glass) and PLL-coated Petri dish (PLL) were analyzed in this section (Figure 3C, *N* = 5, for each group). We performed Welch's ANOVA due to the significantly different variance among groups assessed by Bartlett's test (*p* = 0.0007). Strong difference between the groups was observed (*p* < 0.0001) and multiple comparisons were evaluated with the Games-Howell test. The latter returned a statistical difference among all the comparisons (*p* = 0.0126 PS vs. glass; *p* = 0.0058 glass vs. PLL; *p* = 0.0052 PS vs. PLL). The same tests were performed for the substrate stiffness, measured at 10 nm (Figure 3D). Significant difference across the groups was confirmed (*p* < 0.0001) and the multiple comparisons showed no statistical difference between the PS and PLL group, albeit significant difference between glass and the other two groups (*p* = 0.455 PS vs. PLL; *p* = 0.0062 PS vs. glass; *p* = 0.0058 glass vs. PLL).

We therefore proceeded to compare the elastic modulus values calculated for the different cell groups. The PLL group showed the lowest Young's modulus (0.5151 ± 0.24 kPa), followed by PS samples (6.323 ± 1.273 kPa) and glass ones (13.4 ± 3.050 kPa). The increased stiffness between glass and PS samples was correlated with the increased stiffness of the substrate (9.545 ± 2.1 and 1.59 ± 0.16 MPa respectively), whereas the measured PLL stiffness was similar to the PS one (1.88 ± 0.47 MPa).

### Effect of genetic manipulation

**Figure 4:**
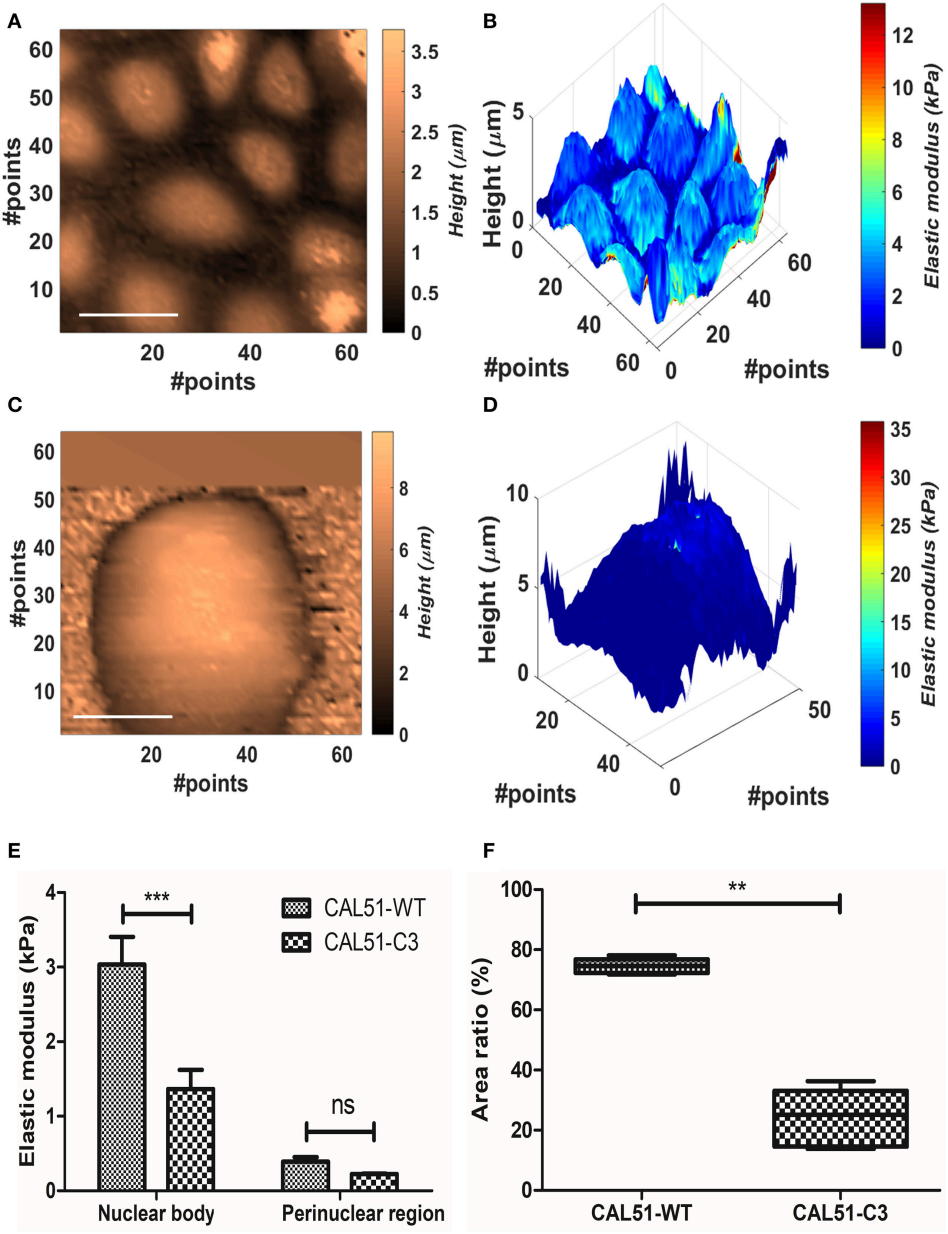
Analysis of the YAP mutation effect on cell mechanics. **(A,B)** Contact point and stiffness tomography maps showing topographical and biomechanical distribution of CAL51-WT cells. Scalebar is 30 μm. **(C,D)** Contact point and stiffness tomography maps of CAL51-C3 colonies. The cells pass from monolayer distribution to spheroidal aggregation due to lack of focal adhesions. Scalebar is 30 μm. **(E)** Bar plot showing elastic modulus differences between two identified regions with different levels of elasticity nuclear and perinuclear, (mean ± SEM). Statistical difference was obtained with two-way ANOVA with Bonferroni posttest (ns, non-significant; ***p < 0.001). **(F)** Boxplot showing the difference in area fraction covered by the nuclear body (line represents the median and whiskers indicate data range). The significant reduction in the mutated samples can reflect the nuclear shape change due to cytoskeletal instability. Statistical difference is obtained using Student's *t*-test with Welch's correction (**p < 0.001).

We measured YAP-deficient cell line CAL51-C3 in comparison with healthy control (CAL51-WT). The cellular samples typically grew in colonies, and this made impossible to identify univocally the position of the substrate. Therefore, Oliver-Pharr's model, which presented the higher stability after the BECC one, was used to calculate the samples elastic modulus, at 300 nm indentation. We compared separately the contribution of harder parts (referred further as nuclear bodies) and softer parts (perinuclear regions). The names were chosen after correlation with topographical images. CAL51-WT samples showed multiple cells per map, organized in a monolayer fashion (Figures 4A,B); conversely, CAL51-C3 aggregated and showed a pronounced spheroidal shape, with hardly distinguishable single cells (Figures 4C,D). Two-way ANOVA was used to estimate if the YAP-deficiency or the subcellular region significantly affected the stiffness results. Both genetic modification and considered region played significant role in the determination of the Young's modulus (*p* < 0.0001). Bonferroni posttest showed a significant difference between the stiffness of the nuclear regions between the two groups (*p* < 0.001), whereas no statistical difference could be found for the perinuclear regions. We observed an apparently different contribution of the nuclear bodies to the sample stiffness, between the two cellular groups. Thus, we calculated the fraction occupied by the nuclear region in the whole cell area. The obtained values were compared using Student's *t*-test with Welch's correction for uneven variances. The group means resulted significantly different (*p* = 0.0003).

According to these outcomes, we compared the values calculated for the Young modulus and the nuclear area fraction. CAL51-C3 presented a significantly decreased nuclear stiffness (1.36 ± 0.57 kPa, *N* = 5) compared to CAL51-WT (3.04 ± 0.83 kPa, *N* = 5), whilst no difference was observable between the perinuclear regions (0.39 ± 0.13 kPa, WT, vs. 0.23225 ± 0.02018 kPa, C3). These results are shown in Figure 4E. The area occupied by the nuclear region was in percentage higher in the WT samples (74.6 ± 2.485%) compared to the C3 ones (24.1 ± 9.6%). These results are summed up in Figure 4F.

## Discussion

The first question of this methodological work focused on influence of the selected biomechanical model on the evaluation of cellular stiffness. We have observed how the choice among three linearly elastic mechanical models greatly affects the calculated stiffness values, at different indentation levels. As first result, we saw how the Bilodeau's modification of Sneddon's theory leads to the highest estimation of the cell stiffness. This can be explained by the breaking of the assumption of infinitely thick sample, or small indentations (Dimitriadis et al., 2002; Radmacher, 2007) over an adhered cell sample. This effect is accounted in the BECC model (Gavara and Chadwick, 2012), by explicit introduction of the sample height in the calculations, and partially mitigated by including the indentation-dependent area of contact in the Oliver-Pharr's one (VanLandingham et al., 2001). Whilst one could limit the analysis to small indentations, levels above 300 nm have shown generally increased stiffness throughout all the models used. This is most probably due to the measurement noise in proximity of the contact point. The interpretation of the FDCs requires a precise, however indirect (BenÍtez et al., 2013), identification of the point of contact between indenter and samples (Melzak et al., 2010), as small errors can lead to one order of magnitude changes in the calculated elastic modulus (Crick and Yin, 2007). Furthermore, force-indentation relationship is dependent on the junction between the sample and the substrate, to such a way that an adhered sample requires higher force to be indented (Chadwick, 2002). The firm or loose adhesion is a paramount issue in AFM measures, as it was shown in previously reported studies that the simple application of the Hertzian model can lead to artificially low Young's modulus (Dokukin et al., 2013). These issues pose difficulty in limiting uniformly the indentation level on spatially anisotropic materials such as cells. Such anisotropic properties were confirmed by the significant effect of the indentation level selected for the stiffness tomography, and the observed surface heterogeneity of the elasticity maps. For all these considerations, the BECC model seems to be the elective choice, thanks to its observed level of discrimination between hard and soft areas, and higher stability rate at increasing depths. Nevertheless, this model is not applicable when the substrate (i.e., an area with clear contact point and linear repulsive regime) is not clearly identifiable. This is the case of biological samples which typically grow in large colonies or monolayers, such as stem cells or uniform cell lines. In this case, the Oliver-Pharr's method provides the second-best characteristics, according to stability rate. It is worth mentioning that all the considerations discussed so far are relevant for models in which the cell-probe interface is well-defined, such as the one presented. Other more complex models, such as the pericellular brush model for spherical indenters, could help to separate the effect of plasma membrane protein layer from the cellular bulk (Sokolov et al., 2013), limiting at least the uncertainty in the detection of the point of contact.

We then applied the two optimal models to case-specific scenarios, in which cell biomechanics is controlled by the surrounding environment or by gene editing.

Surprisingly, we could not find a previous reported application of BECC model over a whole cell. The effect of the substrate stiffness was assessed using BECC model to interpret the force maps measured on AD-MSC cells grown in FN-coated PS/glass surfaces, or PLL-coated PS surface. The implemented statistical tests showed significant differences between all these three groups. Furthermore, the increasing stiffness between the samples grown on glass compared to the ones grown on PS is correlated with the stiffness of the substrate. This is most probably due to the well-known proportional dependency of diverse cell types' cytoskeletal synthesis and contractility on the substrate rigidity (Solon et al., 2007). AD-MSC have been shown to respond to substrate stiffness alteration, but to the best knowledge of the authors this is the first direct characterization of this effect on AD-MSC with AFM force mapping (Keremidarska-Markova et al., 2017), but to the best knowledge of the authors this is the first direct characterization of this effect on AD-MSC with AFM force mapping. On the contrary, the observed plummeting of the cell stiffness on PLL-coated surfaces did not correlate with the substrate rigidity. This absence of correlation is most probably due to the biochemical alteration of the cellular mechanics, since PLL is able to abrogate focal adhesions formation regardless the substrate stiffness (Nardone et al., 2017). A lack of focal adhesion is directly correlated to inhibited cell contractility (Balaban et al., 2001), which is measured by AFM in stiffness decrease.

The targeted knockout of the *YAP1* gene in CAL51 cell line altered sensibly the cell colonies morphology and biomechanics. We firstly observed how the mutated line presented low attachment, and spheroidal shape of the cell colonies, alternatively spreading in monolayer for WT. We then noticed two distinct distribution peaks among the elasticity values. By selecting these two distribution regions and correlating them with topographical images, we inferred observing harder oval nuclear regions (Dvir et al., 2015) and softer perinuclear regions. We did not refer to the latter as cytoplasmic areas since the cell-cell edge was not detectable by force mapping. Statistical analysis showed that stiffness decrease was significant only in the nuclear areas, whereas no significant difference was found across the perinuclear regions of the two groups. This phenomenon can be explained by the absence of focal adhesions assembly, due to the downregulation of the Hippo mechano-transduction pathway by *YAP1* knockout (Nardone et al., 2017). This pathway is fundamental in the regulation of cytoskeleton (actin, myosin II) stability, and consequently the nuclear stiffening in response to mechanical forces. These biomechanical evidences can additionally be correlated with the surface fraction occupied by the stiffer nuclear region, significantly lower for the mutated line. The decrease of area fraction can be explained with the shape change of the nucleus, elongated by cytoskeletal fibers in spread cells and rounded in non-adhering samples. This 3D change, while preserving the total volume, will be sensed by the AFM as decrease in cross-sectional area.

## Conclusions

We have demonstrated how rationalized methodologies concerning AFM measurements and data interpretation can unveil deeper insights than simple biomechanics. To do so, we have applied classical and refined biomechanical models to biophysically and biochemically-controlled samples. We have discussed how the selected mechanical model significantly affects the final results, and this dependency must be considered. The choice of the mechanical model is ultimately conditioned by the sample geometry and substrate properties. The use of advanced AFM methods, such as stiffness tomography, can reveal important 3D heterogeneities of the cellular samples. Simultaneous use of AFM interpretation techniques on hard and soft samples allows scientists to discriminate between physical and biochemical effects on cell elasticity. The combination of reconstructed topography and elasticity maps can reveal selective mutation-induced cellular changes. We hope this methodological work will help the scientific community in understanding the potential of advanced AFM applications, and the underlying complexity of its interpretation techniques, for the quantitative advanced analysis of biological samples.

## Author contributions

GC designed and programmed the software routine utilized in this study. He also performed data analysis and wrote the results and discussion part of the work. JP conducted the atomic force microscopy experiments and supervised the writing of the manuscript with MP. PS supported AFM experiments and contributed to manuscript preparation. JO and GN designed and produced the cellular samples described. GF supervised and approved the cellular experiments and contributed to the AFM financing.

## Data availability statement

All results obtained this study are included in the manuscript and the supplementary files. The datasets and the raw data can be made available to any qualified researcher under reasonable request.

### Conflict of interest statement

The authors declare that the research was conducted in the absence of any commercial or financial relationships that could be construed as a potential conflict of interest.
