# Supplementary material for: Advanced and Rationalized Atomic Force Microscopy Analysis Unveils Specific Properties of Controlled Cell Mechanics
Source: Front Physiol. 2018 Aug 17;9:1121. doi: 10.3389/fphys.2018.01121 (PMC6107778; doi:10.3389/fphys.2018.01121)
Supplement: Supplementary file 1 [file Table_1.docx]

Supplementary Materials

**Advanced and rationalized atomic force microscopy analysis unveils specific properties of controlled cell mechanics**

**Guido Caluori, Jan Pribyl*, Martin Pesl, Jorge Oliver De la Cruz, Giorgia Nardone5 Petr Skladal, Giancarlo Forte**

*** Correspondence:** Jan Pribyl, Ph.D., pribyl@nanobio.cz

# Detailed description of analysis

The obtained force volume maps were processed in a Matlab R2017b graphic user interface (Mathworks, Natick, Massachussets, USA), using a stepwise procedure, with state-of-the-art algorithms and mechanical models. The basic procedure is as follows, and it is mirrored in the different sections of Supplementary figure 1:

1. The compressed file from the JPK software are extracted and converted in the appropriate unit of measurement by the embedded conversion factors. An FDC is associated to each pixel of the 2D grid forming the force-volume map.
2. The approach part of the curve is detrended by a linear fitting on the non-contact portion (I). The point of contact is selected by a linear-quadratic intersection, using a threshold-based trial point (II)
3. A map of point of contact is obtained, by calculating the point of contact of all approaching FDC across the 2D grid. The sample tilting is eliminated by estimating the 2D trend (tilting place) across the map.
4. The contact portion of each FDC is corrected by the cantilever deflection (I), fitted by the selected appropriate model, and the Young’s modulus at different indentations is calculated (II).

The obtained 3D array of Young’s modulus is then saved in MAT format, and further processed for the necessary statistical analysis.

# Supplementary Tables

## Comparison between mechanical models

Table 1: Summary table of repeated measures two-way ANOVA and Bonferroni posttest for the model effect evaluation

| Table Analyzed | Models comparison |  |  |  |
| --- | --- | --- | --- | --- |
|  |  |  |  |  |
| Two-way RM ANOVA | Matching by rows |  |  |  |
|  |  |  |  |  |
| Source of Variation | % of total variation | P value |  |  |
| Interaction | 4.55 | 0.0101 |  |  |
| Row Factor | 27.78 | 0.0027 |  |  |
| Mechanical Model | 36.35 | P<0.0001 |  |  |
| Subjects (matching) | 23.7207 | P<0.0001 |  |  |
|  |  |  |  |  |
| Source of Variation | P value summary | Significant? |  |  |
| Interaction | * | Yes |  |  |
| Row Factor | ** | Yes |  |  |
| Mechanical Model | *** | Yes |  |  |
| Subjects (matching) | *** | Yes |  |  |
|  |  |  |  |  |
| Source of Variation | Df | Sum-of-squares | Mean square | F |
| Interaction | 8 | 44.60 | 5.575 | 2.989 |
| Row Factor | 4 | 272.5 | 68.13 | 5.855 |
| Mechanical Model | 2 | 356.6 | 178.3 | 95.59 |
| Subjects (matching) | 20 | 232.7 | 11.63 | 6.238 |
| Residual | 40 | 74.61 | 1.865 |  |
|  |  |  |  |  |
| Number of missing values | 0 |  |  |  |
|  |  |  |  |  |
| Bonferroni posttests |  |  |  |  |
|  |  |  |  |  |
| Bilodeau vs Oliver-Pharr |  |  |  |  |
| Row Factor | Bilodeau | Oliver-Pharr | Difference | 95% CI of diff. |
| 100 | 14.18 | 8.469 | -5.710 | -8.406 to -3.013 |
| 200 | 10.48 | 7.181 | -3.296 | -5.992 to -0.5991 |
| 300 | 9.399 | 6.586 | -2.813 | -5.510 to -0.1165 |
| 400 | 8.228 | 6.249 | -1.979 | -4.676 to 0.7172 |
| 500 | 7.615 | 6.114 | -1.502 | -4.198 to 1.195 |
|  |  |  |  |  |
| Row Factor | Difference | t | P value | Summary |
| 100 | -5.710 | 6.610 | P<0.001 | *** |
| 200 | -3.296 | 3.815 | P<0.01 | ** |
| 300 | -2.813 | 3.257 | P < 0.05 | * |
| 400 | -1.979 | 2.292 | P > 0.05 | ns |
| 500 | -1.502 | 1.739 | P > 0.05 | ns |
|  |  |  |  |  |
| Bilodeau vs BECC |  |  |  |  |
| Row Factor | Bilodeau | BECC | Difference | 95% CI of diff. |
| 100 | 14.18 | 9.437 | -4.742 | -7.438 to -2.045 |
| 200 | 10.48 | 5.227 | -5.250 | -7.946 to -2.553 |
| 300 | 9.399 | 3.224 | -6.174 | -8.871 to -3.478 |
| 400 | 8.228 | 2.677 | -5.552 | -8.248 to -2.855 |
| 500 | 7.615 | 2.727 | -4.888 | -7.584 to -2.191 |
|  |  |  |  |  |
| Row Factor | Difference | t | P value | Summary |
| 100 | -4.742 | 5.490 | P<0.001 | *** |
| 200 | -5.250 | 6.078 | P<0.001 | *** |
| 300 | -6.174 | 7.148 | P<0.001 | *** |
| 400 | -5.552 | 6.427 | P<0.001 | *** |
| 500 | -4.888 | 5.659 | P<0.001 | *** |
|  |  |  |  |  |
| Oliver-Pharr vs BECC |  |  |  |  |
| Row Factor | Oliver-Pharr | BECC | Difference | 95% CI of diff. |
| 100 | 8.469 | 9.437 | 0.9678 | -1.729 to 3.664 |
| 200 | 7.181 | 5.227 | -1.954 | -4.651 to 0.7423 |
| 300 | 6.586 | 3.224 | -3.361 | -6.058 to -0.6647 |
| 400 | 6.249 | 2.677 | -3.572 | -6.269 to -0.8758 |
| 500 | 6.114 | 2.727 | -3.386 | -6.083 to -0.6898 |
|  |  |  |  |  |
| Row Factor | Difference | t | P value | Summary |
| 100 | 0.9678 | 1.120 | P > 0.05 | ns |
| 200 | -1.954 | 2.262 | P > 0.05 | ns |
| 300 | -3.361 | 3.891 | P<0.01 | ** |
| 400 | -3.572 | 4.136 | P<0.001 | *** |
| 500 | -3.386 | 3.920 | P<0.01 | ** |

Table 2: Summary table of repeated measures one-way ANOVA and Bonferroni posttest for the whole-volume model effect evaluation

| Table Analyzed | Model Comparison - Whole volume |  |  |  |  |
| --- | --- | --- | --- | --- | --- |
|  |  |  |  |  |  |
| Repeated Measures ANOVA |  |  |  |  |  |
| P value | P<0.0001 |  |  |  |  |
| P value summary | *** |  |  |  |  |
| Are means signif. different? (P < 0.05) | Yes |  |  |  |  |
| Number of groups | 3 |  |  |  |  |
| F | 71.79 |  |  |  |  |
| R squared | 0.7495 |  |  |  |  |
|  |  |  |  |  |  |
| Was the pairing significantly effective? |  |  |  |  |  |
| R squared | 0.5150 |  |  |  |  |
| F | 8.476 |  |  |  |  |
| P value | P<0.0001 |  |  |  |  |
| P value summary | *** |  |  |  |  |
| Is there significant matching? (P < 0.05) | Yes |  |  |  |  |
|  |  |  |  |  |  |
| ANOVA Table | SS | df | MS |  |  |
| Treatment (between columns) | 356.6 | 2 | 178.3 |  |  |
| Individual (between rows) | 505.2 | 24 | 21.05 |  |  |
| Residual (random) | 119.2 | 48 | 2.483 |  |  |
| Total | 981.0 | 74 |  |  |  |
|  |  |  |  |  |  |
| Bonferroni's Multiple Comparison Test | Mean Diff. | t | Significant? P < 0.05? | Summary | 95% CI of diff |
| Bilodeau vs Oliver-Pharr | 3.060 | 6.865 | Yes | *** | 1.954 to 4.166 |
| Bilodeau vs BECC | 5.321 | 11.94 | Yes | *** | 4.215 to 6.427 |
| Oliver-Pharr vs BECC | 2.261 | 5.073 | Yes | *** | 1.155 to 3.367 |

## Effect of the substrate

Table 3: Summary table of Welch's ANOVA and Games-Howell posttest for the substrate effect evaluation on cellular stiffness

| Table Analyzed | Sample E |
| --- | --- |
| Welch’s ANOVA | p<0.0001 |
| Test | Games-Howell |
| Comparisons | p-value |
| PS vs Glass | 0.0126 |
| Glass vs PLL | 0.0058 |
| PS vs PLL | 0.0052 |

Table 4: Summary table of Welch's ANOVA and Games-Howell posttest for the different substrate stiffness

| Table Analyzed | Sample Substrate |
| --- | --- |
| Welch’s ANOVA | p<0.0001 |
| Test | Games-Howell |
| Comparisons | p-value |
| PS vs Glass | 0.0062 |
| Glass vs PLL | 0.0058 |
| PS vs PLL | 0.4554 |

## Effect of the genetic modification

Table 5: Summary table of two-way ANOVA and Bonferroni posttest for the genetic modification and regional effect on cell stiffness

| Table Analyzed | Genetic modification |  |  |  |
| --- | --- | --- | --- | --- |
|  |  |  |  |  |
| Two-way ANOVA |  |  |  |  |
|  |  |  |  |  |
| Source of Variation | % of total variation | P value |  |  |
| Interaction | 9.72 | 0.0045 |  |  |
| YAP-Deficiency | 14.53 | 0.0010 |  |  |
| Position | 61.49 | P<0.0001 |  |  |
|  |  |  |  |  |
| Source of Variation | P value summary | Significant? |  |  |
| Interaction | ** | Yes |  |  |
| YAP-Deficiency | *** | Yes |  |  |
| Position | *** | Yes |  |  |
|  |  |  |  |  |
| Source of Variation | Df | Sum-of-squares | Mean square | F |
| Interaction | 1 | 2.827 | 2.827 | 10.91 |
| YAP-Deficiency | 1 | 4.224 | 4.224 | 16.30 |
| Position | 1 | 17.88 | 17.88 | 69.00 |
| Residual | 16 | 4.146 | 0.2591 |  |
|  |  |  |  |  |
| Number of missing values | 0 |  |  |  |
|  |  |  |  |  |
| Bonferroni posttests |  |  |  |  |
|  |  |  |  |  |
| CAL51-WT vs CAL51-C3 |  |  |  |  |
| Position | CAL51-WT | CAL51-C3 | Difference | 95% CI of diff. |
| Nuclear body | 3.035 | 1.364 | -1.671 | -2.467 to -0.8750 |
| Perinuclear region | 0.3921 | 0.2249 | -0.1672 | -0.9633 to 0.6289 |
|  |  |  |  |  |
| Position | Difference | t | P value | Summary |
| Nuclear body | -1.671 | 5.191 | P<0.001 | *** |
| Perinuclear region | -0.1672 | 0.5195 | P > 0.05 | ns |

Table 6: Summary table of Student's t-test with Welch's correction for the genetic modification effect on measured nuclear area fraction

| Table Analyzed | Area ratio |
| --- | --- |
| Column A | CAL51-WT |
| Vs | vs |
| Column B | CAL51-C3 |
|  |  |
| Unpaired t test with Welch's correction |  |
| P value | 0.0003 |
| P value summary | *** |
| Are means signif. different? (P < 0.05) | Yes |
| One- or two-tailed P value? | Two-tailed |
| Welch-corrected t, df | t=11.38 df=4 |
|  |  |
| How big is the difference? |  |
| Mean ± SEM of column A | 74.56 ± 1.109 N=5 |
| Mean ± SEM of column B | 24.09 ± 4.293 N=5 |
| Difference between means | 50.46 ± 4.433 |
| 95% confidence interval | 38.16 to 62.77 |
| R squared | 0.9701 |
|  |  |
| F test to compare variances |  |
| F,DFn, Dfd | 14.99, 4, 4 |
| P value | 0.0225 |
| P value summary | * |
| Are variances significantly different? | Yes |
