# Supplementary figures and images for: Advanced and Rationalized Atomic Force Microscopy Analysis Unveils Specific Properties of Controlled Cell Mechanics
Source: Front Physiol. 2018 Aug 17;9:1121. doi: 10.3389/fphys.2018.01121 (PMC6107778; doi:10.3389/fphys.2018.01121)

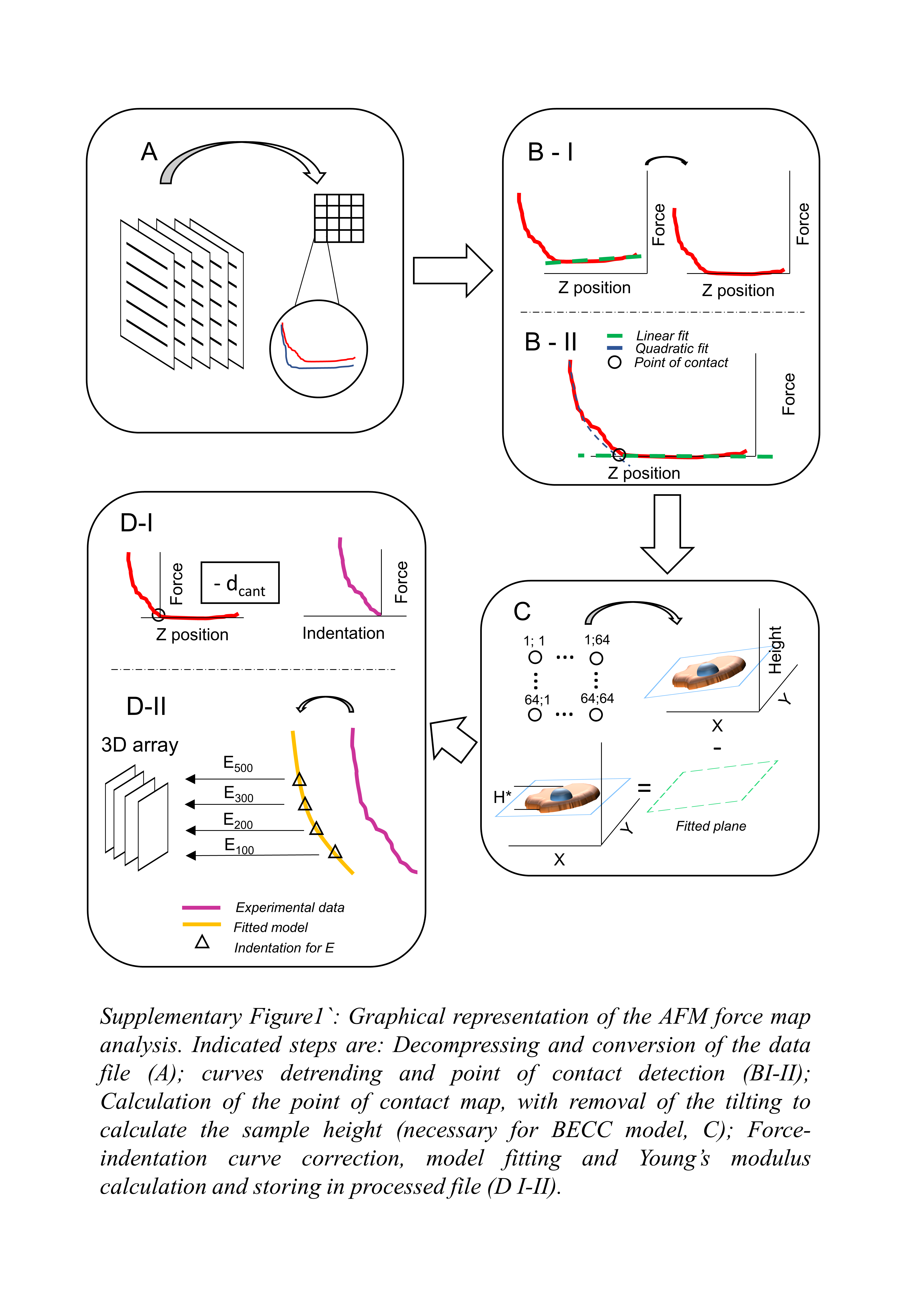

Supplement: Supplementary file 2 [file Image_1.tif]

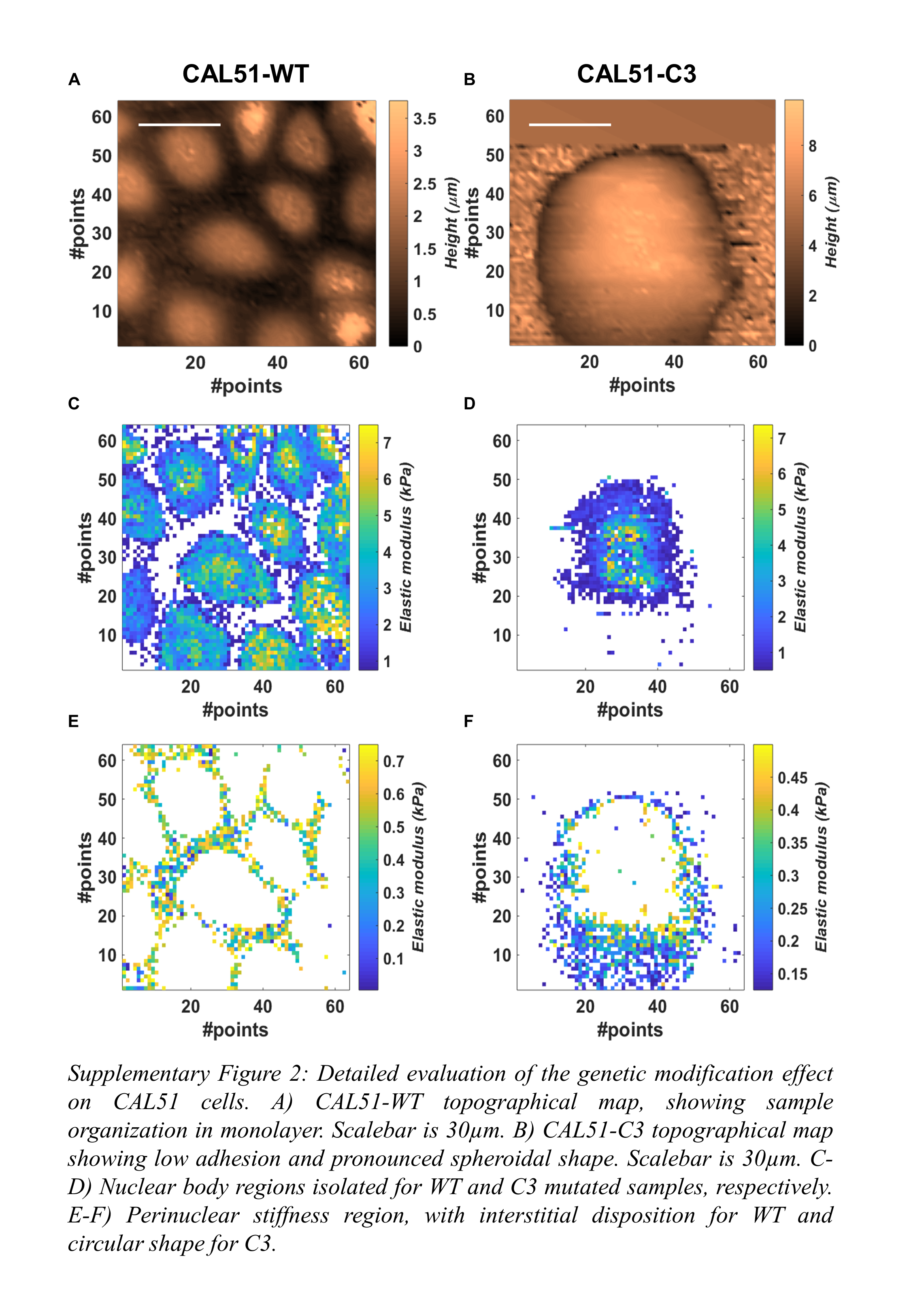

Supplement: Supplementary file 3 [file Image_2.tif]
